# Flexible and Gas-Resistant Films Based on Cellulose Nanofiber and Poly(butylene adipate-*co*-terephthalate)

**DOI:** 10.3390/molecules31030464

**Published:** 2026-01-29

**Authors:** Tingwei Deng, Yaoting Liang, Tong Luo, Feiyun Li, Yanjun Tang

**Affiliations:** College of Textile Science and Engineering, Zhejiang Sci-Tech University, Hangzhou 310018, China

**Keywords:** cellulose nanofiber, PBAT, flexible film, gas-resistant film

## Abstract

Cellulose nanofiber (CNF) has attracted increasing attention as a sustainable nanomaterial for high-performance films due to its renewability and outstanding mechanical properties. However, the practical applications of CNF films are largely hindered by their insufficient tensile flexibility and gas barrier performance. In the present work, a reinforced, multifunctional nanocomposite film was prepared via the solution casting method by incorporating CNF with poly(butylene adipate-*co*-terephthalate) (PBAT). The influence of PBAT loading on the mechanical flexibility and barrier performance of the nanocomposite film was investigated, and the interfacial bonding characteristics were also studied. As a result, the composite film containing 40 wt% PBAT (denoted as CNF-PBAT40) exhibited a tensile strength of 49.6 MPa, which is generally seven times higher than that of the pristine CNF film. Moreover, its flexibility was notably enhanced, reaching an elongation at break of 7.8%. Additionally, the CNF-PBAT40 composite film showed a markedly reduced air permeability of 2.6 μm·Pa^−1^·s^−1^, compared with 9.5 μm·Pa^−1^·s^−1^ for the pristine CNF film. Therefore, these synergistically enhanced properties render CNF-PBAT composite films promising candidates for advanced applications in next-generation sustainable packaging.

## 1. Introduction

The growing environmental crisis and plastic pollution have driven the pursuit of biodegradable alternatives to conventional synthetic polymers in scientific and industrial research [1]. Among the alternatives, cellulose nanofiber (CNF) is an advanced biomaterial made mainly from renewable forest and agricultural resources, which has shown great promise in diversified application fields [2]. Specifically, due to its outstanding mechanical properties, high specific surface area, good biocompatibility, and strong biodegradability [3], CNF has emerged as a promising candidate for a wide range of applications, e.g., sustainable packaging [4], biomedical products [5,6,7,8], and electronic devices [9]. Nevertheless, the practical utilization of CNF films remains limited because of inherent shortcomings, particularly their low tensile flexibility, poor elongation at break, and insufficient gas barrier performance, which compromise their functionality in demanding environments, e.g., food packaging and electronic encapsulation.

To overcome the above limitations, various strategies have been devoted to the chemical modification and the blending process to improve the flexibility and gas barrier properties of CNF-based materials. For instance, the interfacial bonding between CNF and the polymer matrix has been enhanced through acetylation [10] or poly(3-hydroxybutyrate-co-3-hydroxyvalerate) (PHBV) grafting [11]. However, these approaches often involve complex synthesis procedures, elevated production costs, and potential secondary environmental hazards [12]. Although the melt blending methods improved tensile strength, when 7 wt% CNF was incorporated into poly(butylene succinate) (PBS) [13], the high-temperature shear forces during processing often disrupt CNF nanostructure, resulting in reduced flexibility [14]. Alternative strategies, such as incorporating nanofillers (e.g., nano-SiO_2_ or nano-lignin), can improve the barrier properties of CNF films, but often at the expense of mechanical toughness [15]. Moreover, the polymer matrices applied in these processes basically lack sufficient interfacial compatibility with CNF, further limiting their effectiveness. Consequently, an ideal matrix that can achieve strong interfacial interactions with CNF while maintaining the mild processability and overall performance essentially becomes a very important research task.

Commonly, the strong hydrogen bonding between the ester groups of poly(butylene adipate-co-terephthalate) (PBAT) and the hydroxyl groups of CNF [16,17,18,19] can offer the potential to enhance interfacial compatibility and performance. These features make PBAT a promising matrix for constructing high-performance CNF-based composites via mild processing techniques. For instance, PBAT/CNF composites can enhance mechanical properties through an in situ fibrillation strategy [20]. However, the inhomogeneous dispersion of CNF within the network generally hinders molecular-level interactions, thus leading to the limited barrier performance. As a consequence, it is crucial to develop rational strategies to improve the molecular-level interactions between CNF and PBAT to achieve mechanical robustness, enhanced barrier properties, and green processability.

**Figure 1:**
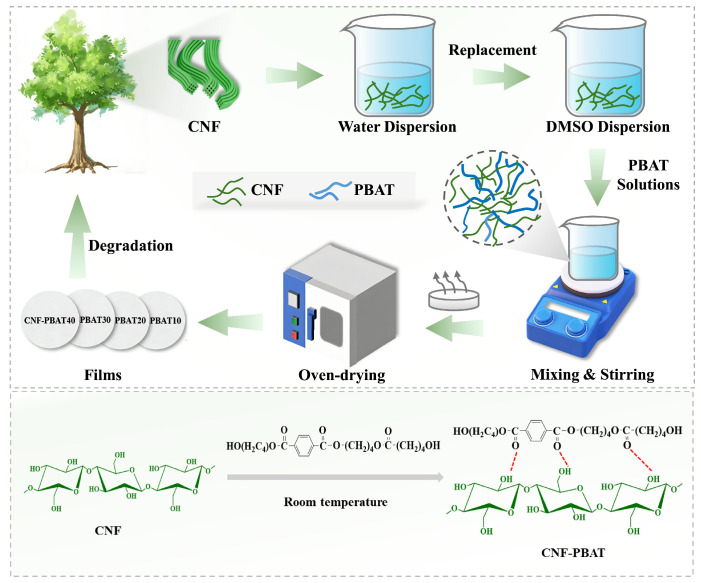
Schematic illustration of the preparation of CNF-PBAT nanocomposite films.

To address these challenges, this work proposes a hydrogen bonding strategy that enables synergistic enhancement of multifunctional properties without resorting to chemical modification (Figure 1). CNF was integrated with PBAT based on a homogeneous system, enabling uniform dispersion and hydrogen bonding between molecular chains, thereby establishing strong interfacial compatibility. This enhanced interfacial bonding was engineered through optimization, eliminating exogenous compatibilizers and avoiding the thermal degradation associated with melt blending. Subsequently, the resultant CNF-PBAT composite film was obtained via the solution casting method, which exhibited a remarkable synergistic effect between mechanical toughness (enhanced strength and flexibility) and gas barrier performance, alongside improved hydrophobicity. Overall, this work offers an effective solution to the preparation of high-performance cellulose-based films, which might hold great promise in biodegradable packaging application.

## 2. Results and Discussion

### 2.1. Rheological Characteristics of CNF Composite Suspensions

**Figure 2:**
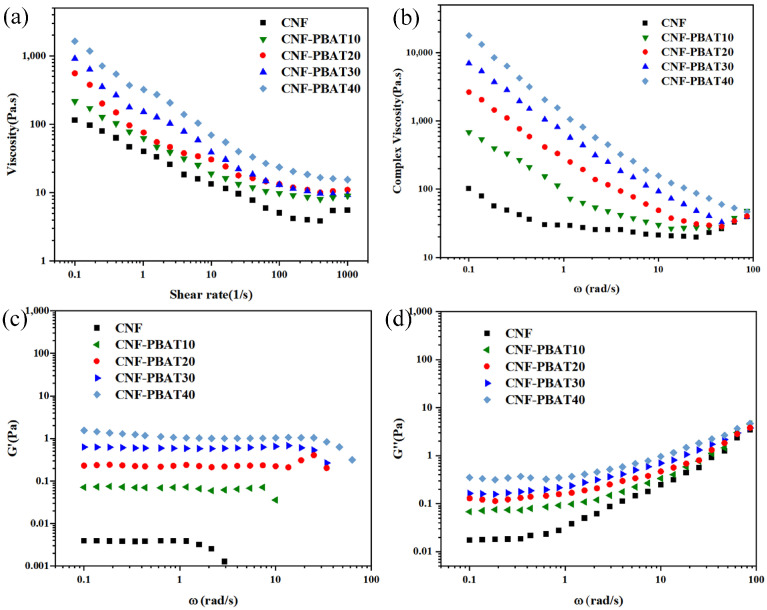
Rheological properties of CNF and CNF-PBAT suspensions. (**a**) Steady-state viscosity (*η*) versus shear rate (*γ*), (**b**) complex viscosity (η*), (**c**) storage modulus (*G*′), and (**d**) loss modulus (*G*″) as functions of angular frequency (*ω*).

The homogeneous dispersion and structural stability of CNF and CNF-PBAT composite suspensions were evaluated by rheological measurements. As shown in Figure 2a, a continuous decrease in viscosity was observed for CNF and CNF-PBAT composite suspensions as the shear rate increased from 0.01 to 100 s^−1^. This shear-thinning behavior was attributed to the alignment of CNF and disentanglement of PBAT chains under high shear forces, which reduced flow resistance [21,22,23]. The complex viscosity (η*) of all suspensions was also gradually reduced with increasing angular frequency from 0.01 to 100 rad/s (Figure 2b), due to the structural relaxation caused by high-frequency shear [24].

The *G*′ of CNF suspensions and CNF-PBAT solutions remained stable at low frequencies and progressively decreased with rising angular frequency (Figure 2c). This trend was explained by the dominance of elastic networks at low frequencies, where the entangled structures formed by CNF and PBAT chains remained intact. Above a critical angular frequency, the chain disentanglement or fiber breakage occurred, leading to reduced elastic recovery capability [25,26]. In contrast, the *G*″ of all systems continuously increased with angular frequency (Figure 2d), indicating enhanced frictional interactions between molecular chains and fibers at elevated frequencies [27]. Notably, the viscosity, η*, *G*′, and *G*″ were progressively enhanced with increasing PBAT content compared to pure CNF. This enhancement was explained by the entanglement network formed through van der Waals forces between PBAT chains and CNF. In addition, the hydrogen bonding between hydroxyl groups on CNF surfaces and ester groups of PBAT further promotes uniform dispersion and stabilizes the composite network [28].

### 2.2. Morphology of CNF Composite Films

**Figure 3:**
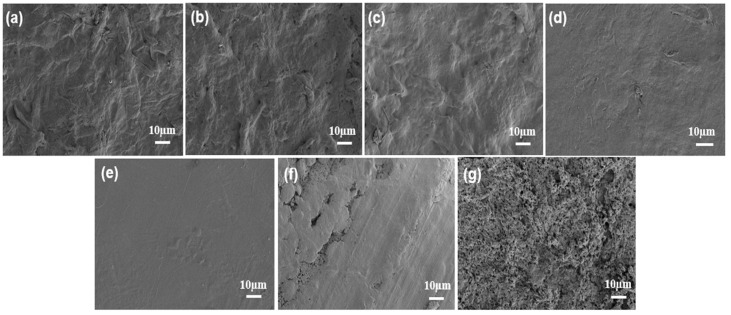
SEM of the surface of (**a**) CNF, (**b**) CNF-PBAT10, (**c**) CNF-PBAT20, (**d**) CNF-PBAT30, (**e**) CNF-PBAT40, (**f**) CNF-PLA20 and (**g**) CNF-PBS20.

FE-SEM was employed to systematically investigate the surface and cross-sectional morphology of CNF-based composite films. As shown in Figure 3a, the CNF film exhibited characteristic rough and porous fibrous network structures. As the PBAT content increased from 10% to 40%, the surface of the CNF-PBAT composite films transitioned gradually from rough to smooth. At a low PBAT content of 10% (Figure 3b), PBAT was dispersed as discrete structures within the CNF network and failed to fully cover the porous skeleton formed by cellulose fibers, leaving evident fibrillar pores and undulating surface morphology. When the PBAT content was increased to 20–30% (Figure 3c,d), PBAT began to form a more continuous phase, effectively wetting and encapsulating the CNF fibers. This resulted in pore filling and a reduction in surface roughness. At 40% PBAT content (Figure 3e), a sufficiently continuous PBAT matrix was formed, which completely covered the CNF network. Consequently, surface pores were nearly eliminated, yielding a uniform and dense film surface. Comparatively, a uniform morphology characterized by the absence of cracks or pores was exhibited in CNF-PLA20 (Figure 3f), whereas distinct micron-scale voids were observed in CNF-PBS20 (Figure 3g), accompanied by structural looseness.

This morphological evolution suggests the potential for enhanced structural integrity and a more continuous barrier architecture. Interfacial compatibility was enhanced through hydrogen bonding and physical entanglement by PBAT, accompanied by CNF orientation being induced via long-chain penetration. While polylactic acid (PLA) achieved some degree of compatibility via polar interactions, its rigid molecular structure and lack of hydrogen bonding led to limited interfacial integration with CNF. For CNF-PBS composite films, severe phase separation occurred due to the mismatch in chain rigidity between CNF and PBS. The flexible aliphatic chains of PBS were incompatible with the rigid CNF, leading to poor dispersion and the formation of distinct phase domains.

### 2.3. FT-IR Analysis of CNF Composite Films

**Figure 4:**
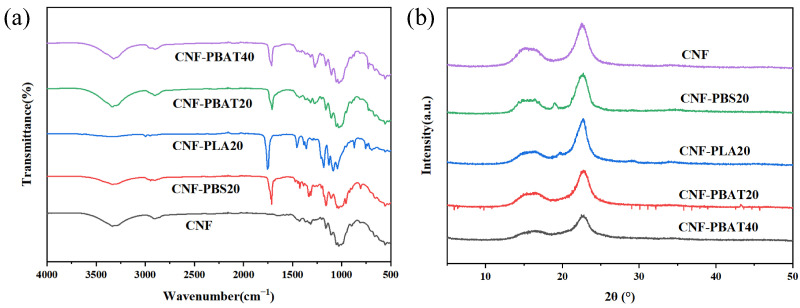
(**a**) FT-IR spectra and (**b**) XRD pattern of CNF and CNF composite films.

The FT-IR spectra of CNF films and composite films were presented in Figure 4a. Characteristic peaks associated with CNF were observed at 3330 cm^−1^ (O-H stretching vibration) and 1030 cm^−1^ (C-O stretching vibration). The peak at 1452 cm^−1^ corresponds to aromatic ring skeletal vibrations in CNF. Distinctive peaks were observed at 1754 cm^−1^ (assigned to C=O stretching vibrations of ester groups), 1184 cm^−1^ (attributed to C-O stretching), and 1454 cm^−1^ (corresponding to -CH_3_ bending vibration) in CNF-PLA20. CNF-PBS20 was characterized by absorption bands at 1712 cm^−1^ (C=O stretching), 1160 cm^−1^ (C-O stretching), and 703 cm^−1^ (-CH_2_ rocking mode). In CNF-PBAT composites, the absorption peak at 1712 cm^−1^ was attributed to carbonyl vibrations in ester linkages.

For CNF-PBS20, the C=O peak was observed at 1714 cm^−1^, exhibiting a slight redshift. It was attributed to the rigidity and low polarity of the PBS chains, which allowed only weaker hydrogen bonds to form with CNF. In contrast, a local blueshift effect was detected for CNF-PLA20, with its C=O peak appearing at 1726 cm^−1^. This change was mainly ascribed to the high crystallinity and steric hindrance of PLA, which limited the number of hydrogen bonds and suppressed the formation of strong, effective hydrogen bonding in the bulk phase. By comparison, the C=O peak of CNF-PBAT20 was significantly redshifted to 1706 cm^−1^. This indicated that the carbonyl groups of PBAT acted as effective hydrogen bond acceptors, forming strong hydrogen bonds with the O-H groups of CNF. Notably, a progressive red shift in the O–H stretching peaks (3330 and 3320 cm^−1^) appeared in CNF-PBAT samples with increasing PBAT content. This shift confirmed the formation of hydrogen bonds between PBAT ester groups (C=O) and CNF hydroxyls (O–H), resulting in a decrease in free O–H groups and peak intensity. Therefore, such hydrogen bonding interactions not only facilitated PBAT integration into the CNF network but are also expected to reinforce interfacial adhesion, contributing to enhanced mechanical strength [29].

### 2.4. XRD Analysis of CNF Composite Films

The crystal structures of CNF and CNF-composite films were further investigated by XRD. As shown in Figure 4b, the characteristic diffraction peaks of cellulose I were detected at 14.8°, 16.6°, and 22.8° in CNF and CNF-composite films. For CNF-PLA films, the persistent peak at 22.8° was attributed to co-crystalline interface formation, where hydrogen bonding between PLA rigid chains and CNF induced heterogeneous nucleation of PLA on CNF surfaces [30].

Crystallinity evolution was not observed in CNF-PBS20, indicating poor interfacial compatibility and a physically blended structure [13,31]. In contrast, the incorporation of intrinsically amorphous PBAT disrupted the crystalline ordering in CNF films, resulting in predominantly amorphous composite structures. The CNF-PBAT composite films showed characteristic red shifts and intensity attenuation in the peaks as PBAT content increased (Figure 4b), reflecting the disruption of intrinsic CNF hydrogen bonding.

### 2.5. Thermal Stability of CNF Composite Films

**Figure 5:**
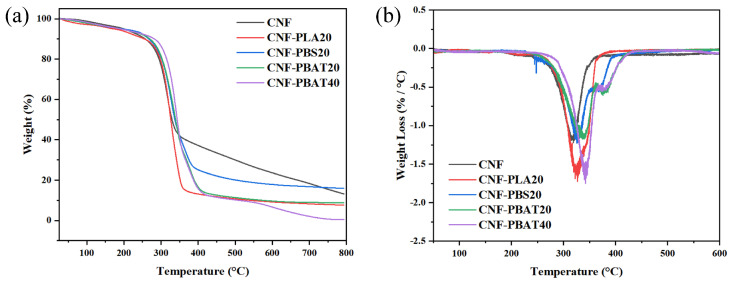
(**a**) TGA curve and (**b**) DTG curve of CNF composite films.

The thermal stability of CNF and CNF composite films was assessed by TGA to investigate the effect of intermolecular interactions on their thermal behavior. As shown in Figure 5a,b, all samples exhibited an initial weight loss of approximately 5% below 200 °C, which was attributed to moisture evaporation and degradation of low-molecular-weight oligomers [32]. The initial decomposition temperature (*T*_0_) and maximum decomposition temperature (*T*_max_) were summarized in Table 1. Compared with pure CNF, the *T*_0_ of CNF-PBAT20 and CNF-PBAT40 was enhanced from 244.63 °C to 255.26 °C while the *T*_max_ was elevated from 339.39 °C to 342.16 °C. These improvements were attributed to hydrogen bonding and the formation of densely packed composite structures, which hindered heat and oxygen diffusion, thereby delaying thermal decomposition chain reactions.

However, compared to PBAT, weaker interfacial bonding in PLA and phase separation induced by the flexible aliphatic chains of PBS led to limited enhancement of thermostability. For CNF-PLA20 and CNF-PBS20, the *T*_0_ was increased to 243.56 °C and 243.97 °C, and *T*_max_ was 326.75 °C and 329.48 °C, respectively. These results indicate that the enhanced thermal stability of CNF/PBAT composite films is attributable to strengthened intermolecular interactions, including hydrogen bonding and improved interfacial compatibility between CNF and PBAT chains.

### 2.6. XPS Analysis of CNF Composite Films

**Figure 6:**
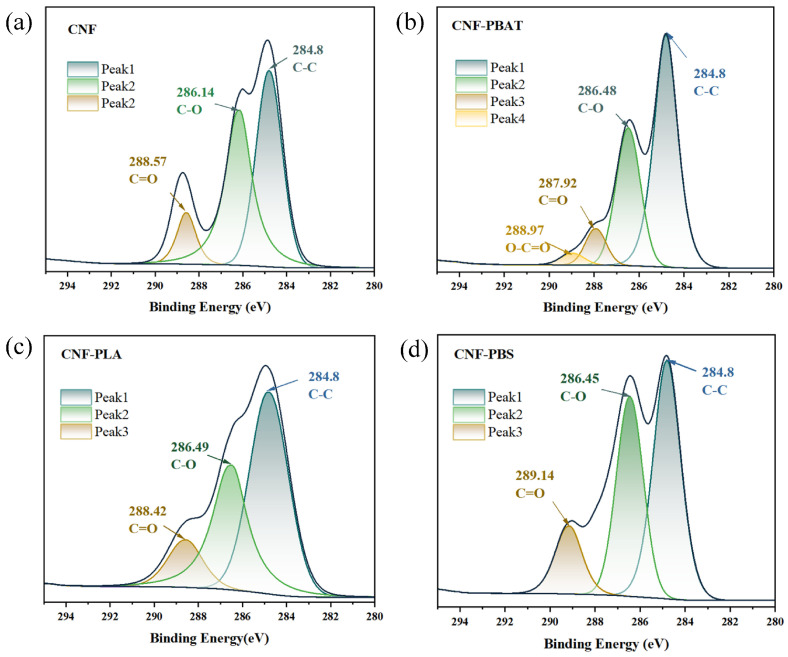
XPS spectra of element distribution of (**a**) CNF, (**b**) CNF-PBAT, (**c**) CNF-PLA, and (**d**) CNF-PBS composite films.

As shown in Figure 6a,b, the C-O bond binding energy was increased from 286.14 eV (CNF) to 286.48 eV (CNF-PBAT20), indicating enhanced physical entanglement between PBAT chains and CNF hydroxyl groups. A negative shift of 0.65 eV in the C=O bond, from 288.57 eV (CNF) to 287.92 eV (CNF-PBAT20), was observed, which demonstrated that hydrogen bonding was formed between PBAT ester groups and CNF hydroxyls. This leads to an increased electron cloud density of the ester oxygen atoms and a consequent decrease in binding energy. As a comparison, Figure 6c,d shows that the ester group (C=O) of CNF-PLA20 was detected at 288.42 eV, corresponding to a small negative shift of −0.15 eV compared to pristine CNF, which confirms dipole–dipole interactions along with hydrogen bonding with CNF hydroxyls. In CNF-PBS20, the C=O binding energy increased to 289.14 eV, representing a positive shift of +0.57 eV. This high binding energy state is maintained because the PBS butanediol segments possess inherent rigidity and low polarity [33], which prevented effective hydrogen bonding or chemical coupling with CNF hydroxyl groups (O-H).

Based on the analysis of the binding energy shifts described above, a clear positive correlation between hydrogen bond strength and composite film performance could be established. A larger negative shift in the C=O binding energy indicated the formation of stronger hydrogen bonds between CNF hydroxyl groups and polyester carbonyl groups. It led to more significant interfacial electron cloud rearrangement and interaction, thereby imparting the composite film with superior interfacial compatibility, improved interfacial adhesion, and enhanced overall properties. Conversely, a positive shift suggested only weak hydrogen bonding, where the interface was dominated mainly by physical entanglement, resulting in limited improvement in the film’s performance. In this series, the hydrogen bond strength followed CNF-PBAT20 > CNF-PLA20 > CNF-PBS20. This quantitative relationship offered direct spectroscopic guidance for designing high-performance CNF-based composite films.

### 2.7. Mechanical Properties of CNF Composite Films

**Figure 7:**
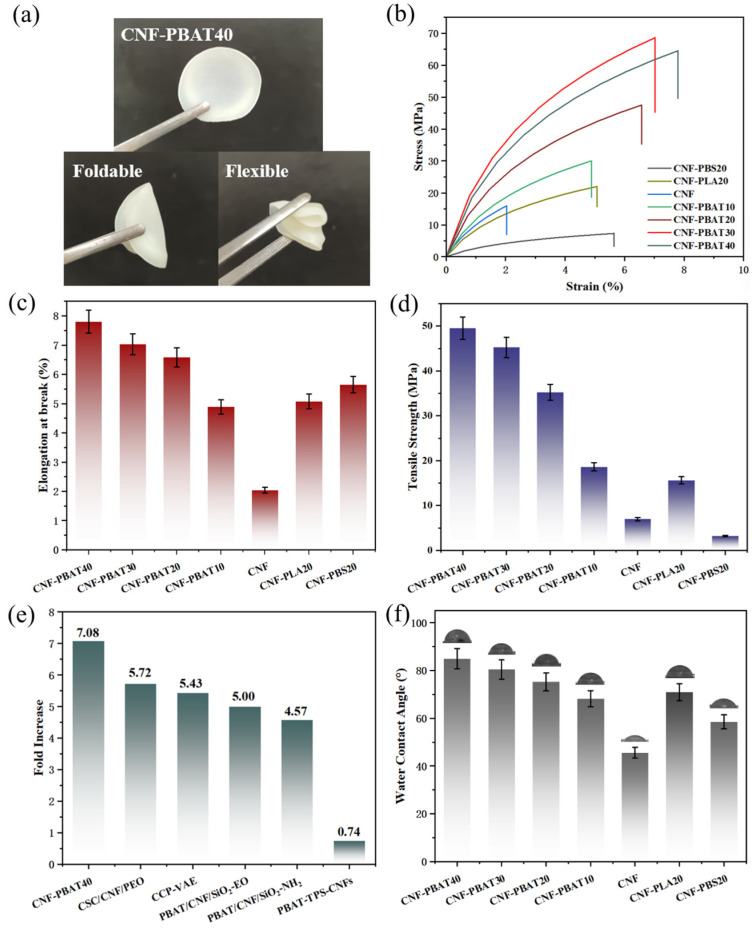
(**a**) Digital photographs of CNF-PBAT40 and their demonstration of mechanical properties; (**b**) Tensile stress–strain curves, (**c**) elongation at break and (**d**) tensile strength of CNF and its composite films; (**e**) comparative analysis of tensile strength and reinforcement efficiency between CNF-PBAT40 and CNF-based composites; (**f**) water contact angle of the CNF composite films.

As demonstrated in Figure 7a, CNF-PBAT40 displayed outstanding mechanical integrity, maintaining structural continuity without any fracture or damage even after repeated folding. Subsequently, the mechanical properties of the CNF composite films were systematically evaluated through uniaxial tensile tests. As shown in Figure 7b,c, the CNF-PBAT composite films exhibited a gradual increase in strain and elongation at break with increased PBAT content. CNF-PBAT40 exhibited an elongation at break of 7.8%, representing a significant increase from 2.04% for pristine CNF films.

CNF/PBAT composite films demonstrated improved softness and bendability, primarily due to the reduced proportion of rigid CNF and the concomitant increase in flexible PBAT segments (Figure 7b,c). In contrast, the tensile strength increased from 7.0 MPa for pristine CNF to 15.6 MPa for CNF-PBAT10, corresponding to a 1.2-fold enhancement (Figure 7d). Moreover, the CNF-PBAT20 exhibited superior mechanical performance with a tensile strength of 35.3 MPa, markedly exceeding that of CNF-PLA20 (15.6 MPa) and CNF-PBS20 (3.3 MPa). This was attributed to the differing interfacial interactions between CNF and polymers. PBAT flexible adipate-derived chains and high-density ester groups promoted hydrogen bonding stress coupling with CNF, where carbonyl oxygen atoms acted as acceptors for CNF hydroxyl protons, forming a reversible crosslinked network that facilitated efficient load transfer. In contrast, the polar ester groups along the PLA chains exhibited limited interfacial interaction with CNF. Moreover, the rigid and brittle molecular backbone restricted chain entanglement and adaptability, resulting in only modest mechanical enhancement. As for PBS, with high crystallinity and low polarity, severe phase separation occurred between PBS and CNF, which ultimately impaired the mechanical strength of the composite. As illustrated in Figure 7d, when the PBAT content increased to 40 wt%, the tensile strength of the CNF-PBAT40 further improved to 49.6 MPa, representing a 7.08-fold enhancement compared to pristine CNF films. CNF-PBAT40 surpassed traditional chemical modification systems (including 5.43-fold for CCP-VAE [34] and 5.00-fold for PRAT/CNSO-CO) by 30% and 42%, respectively. This superiority stemmed from the synergistic effect of CNF’s rigidity and PBAT’s toughness, avoiding the typical strength-toughness/processability trade-off common in conventional chemical approaches. With renewable raw materials and excellent processability, its comprehensive performance is better aligned with industrial and environmental demands (Figure 7e) [35,36,37,38].

### 2.8. Water Contact Angle of CNF Composite Films

The hydrophobicity of CNF-based films was recognized as a critical indicator for their application in food preservation and evaluated via water contact angle measurements (Figure 7f). The water contact angles of CNF films, CNF-PLA20, CNF-PBS20, and CNF-PBAT20 were 45.56°, 70.93°, 58.5°, and 75.23°, respectively, indicating that all three biodegradable polymers contributed to the enhancement of surface hydrophobicity, among which PBAT induced the most substantial increase. Moreover, as the PBAT content increased to 30% and 40%, the water contact angles rose to 80.4° and 84.93°, respectively. This was primarily caused by the reduction of hydroxyl groups on CNF surfaces through hydrogen bonding interactions with hydrophobic aliphatic polyester segments in PBAT molecular chains. In addition, hydrophilic hydroxyl groups of CNF were physically shielded through chain entanglement, thereby lowering polarity and forming a continuous hydrophobic barrier layer [39]. These interactions minimized water adsorption pathways and improved waterproof performance [40], highlighting the potential of PBAT in moisture-sensitive packaging applications.

### 2.9. Gas Barrier Properties of CNF Composite Films

**Figure 8:**
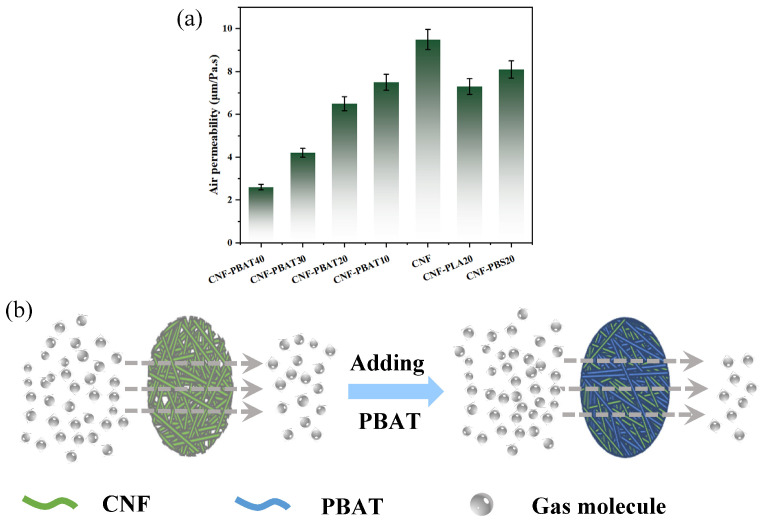
(**a**) Gas barrier properties of CNF composite films; (**b**) Schematic illustration of the gas barrier mechanism in CNF-PBAT composite films.

The air permeability of CNF composite films was systematically investigated to evaluate their gas barrier performance. As shown in Figure 8a, the air permeability of the films decreased from 7.5 to 2.6 μm/(Pa·s) with PBAT content increasing from 10 to 40 wt%. At the same polymer loading (20 wt%), CNF-PBAT20 exhibited better gas barrier performance than CNF-PBS20 and CNF-PLA20.

This improvement was attributed to strong hydrogen bonding between the ester groups (-COO-) of PBAT and the hydroxyl groups (-OH) of CNF, which form a continuous “barrier layer” at the filler–matrix interface that can effectively trap gas molecules and redirect their diffusion pathways [41,42,43]. In addition, the incorporation of PBAT decreased surface roughness and reinforced the structural integrity, resulting in lower internal porosity of the films (Figure 8b) [29,44]. These synergistic effects were absent in PLA/CNF (weak polar interactions) and PBS/CNF (phase-separated structure). More importantly, compared to previous methods involving complex procedures [43,45,46] or toxic additives [47,48], leveraging interfacial hydrogen bonding with PBAT provides a straightforward and efficient means to improve CNF film barrier performance and circumvent the limitations of conventional blending.

Of course, there are two sides to every coin. Regarding sustainability, PBAT is biodegradable but petroleum-derived. In general, the CNF-PBAT composite film could be fully degradable under industrial composting conditions. This offers a feasible solution to the end-of-life environmental issues associated with conventional plastic films. However, it should be noted that complete degradation relies on professional industrial composting facilities. These mainly include appropriate temperature, specific microorganisms, and controlled humidity. Under such conditions, the material can achieve full degradation within several months. Future work should include a full life cycle assessment of this composite, which would be important to strengthen its sustainability claim.

## 3. Experimental Section

### 3.1. Materials

CNF suspension (2.8 wt%) was kindly provided by Hangzhou Research Institute of Chemical Industry, Hangzhou, China. Polylactic acid (PLA) was supplied by Shanghai McLean Biochemical Co., Ltd., Shanghai, China. PBS and PBAT were purchased from Hangzhou Mick Chemical Instrument Co., Ltd., Hangzhou, China. Dimethyl sulfoxide (DMSO, reagent grade, 99%) was purchased from Shanghai Macklin Biochemical Technology Co., Ltd. Shanghai, China, and deionized water was obtained from a laboratory water purification system.

### 3.2. Preparation of CNF Nanocomposite Films

The CNF-PBAT composite film was prepared via the solution casting method, which is a process that is commonly used and highly scalable. In a typical run, the original CNF suspension was first diluted and then centrifuged using a high-speed centrifuge, after which the supernatant was replaced with DMSO. This solvent exchange procedure was performed three times. For each cycle, after centrifugation, the sample was stirred for 30 min and then subjected to ultrasonic dispersion until a homogeneous CNF dispersion in DMSO was achieved. Subsequently, PBAT was dissolved in DMSO under stirring to obtain a 1 wt% solution. The above PBAT solution was then mixed with the prepared CNF suspension and further stirred to form a homogeneous dispersion. The resulting mixture was cast onto a glass plate and air-dried in a fume hood. Afterwards, the film was further dried in a vacuum oven. Eventually, the dried CNF-PBAT composite film was carefully peeled off from the glass plate for further characterization. The thickness of the composite film was controlled at 0.15 mm, approximately.

In comparison, CNF-PLA and CNF-PBS composite films were, respectively, prepared according to the same procedure, except that PBAT was replaced by PLA and PBS, respectively. These films were demonstrated as CNF-XY, where X represents the polymer matrix (PBAT, PBS, and PLA), and Y represents the weight fraction of the polymer matrix in composites (10, 20, 30, and 40).

### 3.3. Characterization

Field emission scanning electron microscopy (FE-SEM, Ultra55, Carl Zeiss AG, Oberkochen, Germany) was employed to observe the surface and cross-section morphology of CNF composite films. The rheological experiments were measured at 20 °C using an MCR 92 rheometer (Anton Paar, Graz, Austria). The viscosity testing was carried out within the shear rate range of 0.01~1000 s^−1^. The storage modulus (*G*′) and loss modulus (*G*″) were investigated by frequency sweep tests.

The Fourier-transform infrared spectroscopy (FT-IR) spectra of all samples were recorded using a Nicolet IS50 spectrometer (Thermo Fisher Scientific, Waltham, MA, USA) to investigate the chemical composition of the CNF composite films. The measurements were conducted within the range of 4000–500 cm^−1^ using the ATR mode, with a resolution of 4 cm^−1^ and 64 scans.

X-ray diffraction (XRD) patterns of CNF and CNF composite films were performed on an ARL XTRA X-ray diffractometer (Thermo Fisher Scientific, Waltham, MA, USA), with Cu Kα radiation between 5°and 50°. The crystallinity index (*CrI*) was determined by the peak height method in terms of Equation (1).

(1) $$\mathrm{CrI}\left(\%\right)=\frac{I_{\left(200\right)}-I_{\mathrm{am}}}{I_{\left(200\right)}\;}\times 100\%$$

where *I*_(200)_ is the maximum diffraction intensity associated with surface areas of crystalline cellulose, and *I_am_* is the diffraction intensity of an amorphous cellulose fraction.

Elemental composition and chemical states of the CNF composites were analyzed based on K-Alpha X-ray photoelectron spectroscopy (XPS, Thermo Fisher Scientific, Waltham, MA, USA).

The thermogravimetric analysis (TGA) of CNF composite films was analyzed using a thermogravimetric analyzer (TGA 5500, TA Instruments, Newcastle, DE, USA). In this case, the test was performed under an N_2_ atmosphere from 25 °C to 800 °C at a temperature increase rate of 10 °C/min.

The mechanical properties of composite films were tested using a universal tester at a stretch rate of 50 mm/min at room temperature (referred to the standard of GB/T 13022-91 Plastics—Determination of tensile properties of films [49]). The sample size was 30 mm × 5 mm, and averages of at least 3 tests were performed on each sample.

The water contact angle of the composite film was tested using the JY-82B video contact angle tester (Chengde DingSheng Test Machine Equipment Co., Ltd., Chengde, China). 2 μL of deionized water was dropped on the films with a micro syringe, and the droplet images were analyzed and tested.

### 3.4. Measurement of Gas Barrier Properties 

The gas barrier properties of composite films were analyzed by air permeability tester (J-TQY10, Sichuan Changjiang Paper Instrument Co., Ltd., Yibin, China), under constant temperature and humidity. The air permeability is calculated by the following Equation (2).

(2) $$\mathrm{Ps}=\frac{V}{∆p\cdot t}$$

where *Ps* is the permeability; *V* is the volume of gas passing through the sample during the measurement time (the volume of water measured in the measuring cylinder), ∆*p* is the pressure difference on both sides of the U-shaped tube, *t* indicates the test time.

## 4. Conclusions

In summary, a rapid method for fabricating biodegradable CNF-PBAT composite films was developed through the solution casting method. This method utilizes molecular-level hydrogen bonding to ensure effective mechanical reinforcement and barrier integrity. Comparative analyses with CNF-PLA and CNF-PBS systems further revealed that CNF-PBAT composite films possess the strongest interfacial interactions among these composites. This is attributed to enhanced component compatibility and dense hydrogen bonding. Consequently, the resulting CNF-PBAT composite films exhibited outstanding mechanical strength, thermal stability, and multifunctional applicability. The tensile strength of the CNF-PBAT composite was improved by 7 times compared to pristine CNF films. It also maintains thermal stability above 300 °C. The synergistic effect of PBAT endowed the composite films with excellent water resistance and gas barrier properties, with a low air permeability of 2.6 μm/(Pa·s) and an increased water contact angle of 84.93°. These results demonstrate that enhanced interfacial hydrogen bonding might lead to the synergistic improvement in mechanical strength, flexibility, and barrier performance. Based on these superior properties, CNF-PBAT composite films hold significant potential for practical applications as high-performance, biodegradable food packaging materials.

## Figures and Tables

**Table 1:** Thermodynamic parameters of CNF and CNF composite films.

Sample	*T*_0_/°C	*T*_max_/°C
CNF	236.75	316.35
CNF-PLA20	243.56	326.75
CNF-PBS20	243.97	329.48
CNF-PBAT20	244.63	339.39
CNF-PBAT40	255.26	342.16

## Data Availability

Data will be made available on request.
